# Human genetic associations of the airway microbiome in chronic obstructive pulmonary disease

**DOI:** 10.1186/s12931-024-02805-2

**Published:** 2024-04-16

**Authors:** Jingyuan Gao, Yuqiong Yang, Xiaopeng Xiang, Huimin Zheng, Xinzhu Yi, Fengyan Wang, Zhenyu Liang, Dandan Chen, Weijuan Shi, Lingwei Wang, Di Wu, Shengchuan Feng, Qiaoyun Huang, Xueping Li, Wensheng Shu, Rongchang Chen, Nanshan Zhong, Zhang Wang

**Affiliations:** 1 Institute of Ecological Sciences, School of Life Sciences, South China Normal University, Guangzhou, Guangdong Province China; 2 State Key Laboratory of Respiratory Disease, National Clinical Research Center for Respiratory Disease, National Center for Respiratory Medicine, Guangzhou Institute of Respiratory Health, The First Affiliated Hospital of Guangzhou Medical University, Guangzhou, Guangdong Province China; 3 The Hong Kong Polytechnic University, Hong Kong, Hung Hom Kowloon China; 4 Department of Obstetrics and Gynecology, The First People’s Hospital of Foshan, Foshan, Guangdong Province China; 5 Department of Pulmonary and Critical Care Medicine, Shenzhen Institute of Respiratory Diseases, Shenzhen People’s Hospital, The Second Clinical Medical College, Jinan University, The First Affiliated Hospital, Southern University of Science and Technology, Shenzhen, Guangdong Province China; 6 Institute of Ecological Sciences, Biomedical Research Center, School of Life Sciences, State Key Laboratory of Respiratory Disease, South China Normal University, Guangzhou, Guangdong Province China

**Keywords:** COPD, Airway microbiome, GWAS, Microbiome-host genetic interaction, Mendelian randomization

## Abstract

Little is known about the relationships between human genetics and the airway microbiome. Deeply sequenced airway metagenomics, by simultaneously characterizing the microbiome and host genetics, provide a unique opportunity to assess the microbiome-host genetic associations. Here we performed a co-profiling of microbiome and host genetics with the identification of over 5 million single nucleotide polymorphisms (SNPs) through deep metagenomic sequencing in sputum of 99 chronic obstructive pulmonary disease (COPD) and 36 healthy individuals. Host genetic variation was the most significant factor associated with the microbiome except for geography and disease status, with its top 5 principal components accounting for 12.11% of the microbiome variability. Within COPD individuals, 113 SNPs mapped to candidate genes reported as genetically associated with COPD exhibited associations with 29 microbial species and 48 functional modules (*P* < 1 × 10^−5^), where *Streptococcus salivarius* exhibits the strongest association to SNP rs6917641 in *TBC1D32* (*P* = 9.54 × 10^−8^). Integration of concurrent host transcriptomic data identified correlations between the expression of host genes and their genetically-linked microbiome features, including *NUDT1*, *MAD1L1* and *Veillonella parvula*, *TTLL9* and *Stenotrophomonas maltophilia*, and *LTA4H* and *Haemophilus influenzae*. Mendelian randomization analyses revealed a potential causal link between *PARK7* expression and microbial type III secretion system, and a genetically-mediated association between COPD and increased relative abundance of airway *Streptococcus intermedius*. These results suggest a previously underappreciated role of host genetics in shaping the airway microbiome and provide fresh hypotheses for genetic-based host-microbiome interactions in COPD.

**Supplementary Information:**

The online version contains supplementary material available at 10.1186/s12931-024-02805-2.

## Introduction

Chronic obstructive pulmonary disease (COPD) is a leading cause of morbidity and mortality worldwide and is manifested by persistent airway inflammation leading to irreversible airflow limitation and impaired lung function [1–3]. Human genetic variation is implicated in COPD, with the alpha-1 antitrypsin deficiency caused by the rare genetic variants in *SERPRINA1* being the current best described genetic abnormality, accounting for 1–2% of the COPD individuals [4]. Recent large-scale genome-wide association studies (GWAS) have revealed additional genetic loci associated with lung function [5, 6], providing insights into the genetic basis of COPD. Through the hitherto largest multi-ancestry GWAS meta-analysis of lung function comprising of 588.452 individuals, Shrine et al. identified 1020 genetic associations from 559 genes enriched in 29 pathways, delineating a comprehensive landscape for variants, genes, proteins and pathways genetically implicated in COPD^6^.

Mounting evidence has revealed a diverse airway microbial ecosystem or microbiome associated with COPD characteristics [7–12]. The airway microbiome interacts with host response, the disruption of which contributes to COPD pathogenesis [13–15]. Multi-omic approaches have been increasingly applied to characterize interactions between the airway microbiome and host response in COPD. By characterizing the 16S rRNA gene-based microbiome and host transcriptome in 574 COPD individuals, Ramsheh et al. identified association between increased *Moraxella* over *Prevotella* and upregulation of pro-inflammatory genes over genes promoting epithelial defence [16]. Through analyzing paired microbiome and metabolomic data in milder stage COPD, Madapoosi et al. showed combined features of microbiome and metabolome in association with lung function and clinical symptoms [17]. In addition to environmental factors such as smoking and medication use that impact the microbiome [18], host genetic variation can be an inherent factor shaping the individualized microbial community [19–26]. Despite evidence showing human genetic association of the microbiota in body sites such as gut and oral cavity [26, 27], there is a paucity of knowledge regarding whether the airway microbiome is associated with human genetics and how the genetic-microbiome interactions may be implicated in COPD.

Metagenomics have been increasingly applied to characterization of the airway microbiome with its capacity in elucidating microbial functional potentials. Through metagenome and metatranscriptome sequencing of upper and lower airway of COPD individuals, Sulaiman et al. showed enrichment of oral commensals in COPD lower airways is associated with upregulation of inflammatory and tumorigenesis markers [28]. Through a combined metagenome, metabolome, host transcriptome and proteome characterization of 99 COPD and 36 healthy individuals, our previous study has revealed the role of *Lactobacillus*-driven tryptophan metabolism and indole-acetic acid, whose depletion results in increased neutrophilic inflammation through IL-22 signaling [13]. In comparison to the gut microbiome, the predominantly high host-to-microbe ratio in airway specimens poses a unique challenge to obtain sufficient information for the microbiota, often necessitating a deep sequencing strategy to achieve adequate microbial coverage after excluding the bulk of human host reads. This limitation, however, may be accompanied with a benefit, as the bulk of the human reads, which were discarded during the microbiome analysis as a common practice, can be recycled to obtain concurrent information on human genetic variations. In this regard, the co-characterization of microbial-host genetic information through airway metagenomics could open up a unique opportunity to assess the association between the airway microbiome and host genetics.

**Fig. 1:**
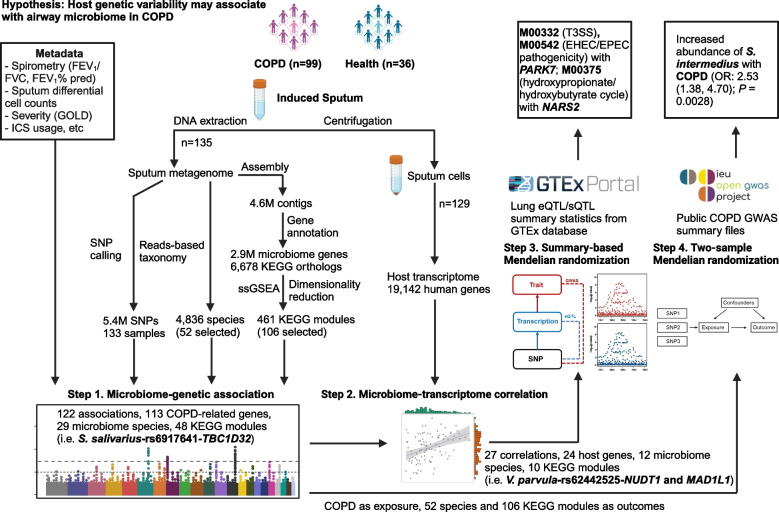
The overall workflow integrating airway metagenomics and host transcriptomics to elucidate airway microbiome-host genetic associations in COPD. Shown are 1) major hypothesis to be tested in the study, 2) the detailed procedure of each step of analysis, 3) the summary statistics of each step, and 4) the potential most promising microbiome-host associations of interest found in each step

Here, we hypothesize that host genetic variation may be associated with the diversity and taxonomic and functional features of the airway microbiome in COPD, and such microbiome-host genetic associations can be captured by airway metagenomic data. By re-analyzing the deeply sequenced sputum metagenomes from 99 COPD individuals and 36 healthy controls (> = 30G sequences per sample) from our previous study [13], we assessed the associations between host genetic variations and the airway microbiome in COPD (Table S1, Fig. 1). We first identified the associations between host single nucleotide polymorphisms (SNPs) and the taxonomic and functional features of the microbiome. Through integrative analysis with concurrent host transcriptomic profiles, we identified the microbiome-host genetic associations that were further transcriptionally linked. We then employed a summary-based Mendelian randomization analysis to identify host genes exhibiting a potential causal association with the microbiome, followed by a bidirectional two-sample Mendelian randomization analysis to refine the causal associations between the microbiome and host genetics. Collectively, these results demonstrates the possibility in analyzing the airway microbiome-host genetic associations through deep metagenomics, providing evidence for host genetic variations that may influence the airway microbiome in COPD.

## Results

### Host genetic variations obtained from the airway metagenomic data

**Fig. 2:**
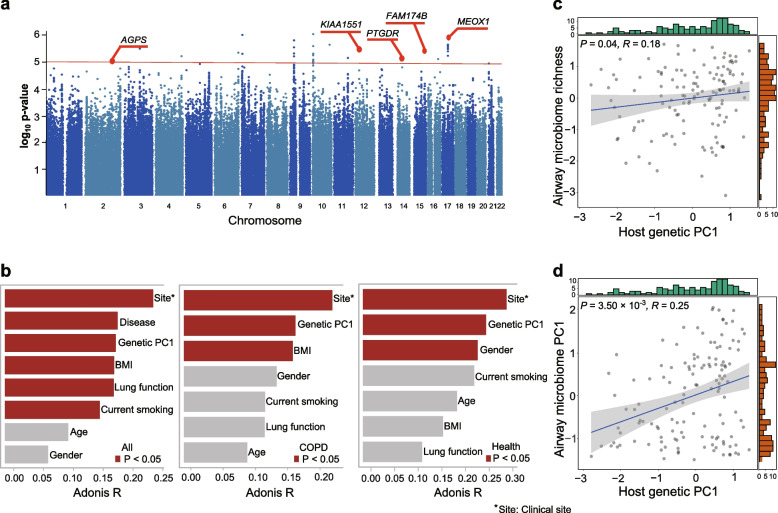
The relationships between host genetics and airway microbial diversity. **a** Manhattan plots of host genetic variants associated with lung function in COPD patients. The red line represents the *P*-value of 1.0 × 10^−5^. Two loci with the significance of genetic association above this threshold and previously identified to be COPD-associated were marked by their gene names (*AGPS* and *PTGDR*). **b** Barplots for the associations of the host genetics and other demographic and clinical variables with the airway microbiome in all the samples and within COPD or healthy individuals as assessed by PERMANOVA. The significant associations (*P* < 0.05) are highlighted in red. **c**, **d** Correlation of the first principal component of host genetic data (x-axis) with (**c**) microbial alpha diversity, and (**d**) the first principal coordinate of the airway microbiome beta diversity (y-axis). The green and red bars are histograms showing the distribution of the X and Y axis

The metagenomic sequencing data yielded an average of 1.83 × 10^8^ high-quality reads per sample, of which an average proportion 91.6% reads are from human host (average ~ 8.4x coverage of human genome) and subject to SNP calling (Fig. S1a). A set of 5,471,650 high quality SNPs were generated. Two of the 135 samples were outliers in principal component analysis and excluded from downstream analyses (Fig. S1b), likely due to their markedly higher missing rate of SNPs compared with the remaining samples (see Methods, Fig. S1c). Density plot showed an even distribution of the SNPs across 22 autosomes with no clear regional preferences (Fig. S1d). When plotting the number of SNPs as a function of the number of individuals sharing a SNP^29^, we observed a rapid decreasing trend of the number of SNPs with the increase of individuals sharing a SNP (Fig. S2), indicating a reasonable genetic variability across individuals in our cohort. The common variants (minor allele frequency > 0.05) were associated with lung function measurement (FEV_1_/FVC) both among all individuals (*n* = 133) and within COPD patients (*n* = 97) using a linear mixed model. Among all individuals, 60 SNPs were identified in association with FEV_1_/FVC involving 10 genomic loci mapped to 6 genes (*P* < 1 × 10^−5^, as suggestive significance, Table S2). Within COPD individuals, 73 SNPs were identified in association with FEV_1_/FVC involving 14 gene loci mapped to 5 genes (Fig. 2a, Table S2). Annotation of these 5 genes in existing functional databases (Open Targets Genetics, GWAS Catalog, and GWASATLAS) revealed two genes (*AGPS* and *PTGDR*) as genetically associated with lung function [29].

We performed additional genetic association analyses using COPD/healthy and FEV_1_% predicted as traits. No SNPs were identified as associated with the binary trait of COPD/healthy (*P* < 1.0 × 10^−5^). Among all individuals, 292 SNPs were identified in association with FEV_1_% predicted involving 22 genomic loci mapped to 18 genes (Table S2, *P* < 1.0 × 10^−5^), with SNP rs16836069 mapped to gene *CSMD2* reaching genome-wide significance (*P* < 5.0 × 10^−8^). Within COPD individuals, 51 SNPs were identified in association with FEV_1_% predicted involving 11 genomic loci mapped to 6 genes (*P* < 1.0 × 10^−5^). No SNPs reached genome-wide significance.

We also performed genetic association analyses with key demographic and clinical parameters, including inflammatory endotype features (sputum neutrophil, eosinophil percentages), severity (Global Initiative for Chronic Obstructive Lung Disease [GOLD] status), and inhaled corticosteroid (ICS) usage and smoking status (Table S2). For neutrophil, 1308 SNPs involving 85 loci mapped to 43 genes were identified (*P* < 1 × 10^−5^), where rs74555247 (mapped to *SFMBT1*) and rs1003669 (mapped to *OXR1*) reached genome-wide significance (*P* < 5 × 10^−8^). For eosinophil, 1550 SNPs involving 114 loci mapped to 70 genes were identified (*P* < 1 × 10^−5^), where rs75059289, rs115876665, rs2277122 (mapped to *ABCC10*), rs356041 (mapped to *PITPNM3*), rs75177701 (mapped to *PIEZO2*) reached genome-wide significance (*P* < 5 × 10^−8^). For GOLD status, 131 SNPs involving 15 loci mapped to 8 genes were identified (*P* < 1 × 10^−5^). For ICS usage, 215 SNPs involving 16 genomic loci mapped to 18 genes were identified (*P* < 1 × 10^−5^), For smoking, 149 genes involving 16 genomic loci mapped to 14 genes were identified (*P* < 1 × 10^−5^). None of the SNPs associated with GOLD, ICS and smoking reached genome-wide significance (*P* < 5 × 10^−8^).

### Host genetic variations are associated with the airway microbial diversity

Permutational multivariate analysis of variance (PERMANOVA) revealed a significant association between host genetic variations and the microbiome composition among all individuals (*P* = 0.001), and within COPD or healthy individuals, respectively (Fig. 2b). When plotting the top 20 principal components (PCs) for host genetics, we found that the slope of the curve generally leveled off after the first PC, suggest the first PC (PC1) could be relatively informative (Fig. S3). Among all individuals, host genetic PC1 was ranked the third among all demographic and clinical features tested in association with the microbiome following site and disease status (COPD or health), accounting for 3.46% of the microbiome variation. When considering the top 5 host genetic PCs, 12.11% of the microbiome variance can be explained. Within COPD or healthy individuals, host genetic PC1 exhibited the greatest association with the microbiome among all features except for clinical site (Fig. 2b, *P =* 0.005 and *P* = 0.008). A significant correlation was observed between host genetic PC1 and both microbial alpha (richness, R = 0.18, *P* = 0.040, Fig. 2c) and beta diversity (the first principal coordinate using Bray-Curtis dissimilarity matrix, *R* = 0.25, *P* = 0.0035, Fig. 2d), indicating a close association between host genetics and the airway microbial diversity.

We also performed an additional PERMANOVA by using microbiome PC1 (explaining 23.3% of the taxonomic diversity) and demographic and clinical variables to associate with the host genetic profiles. Among all 133 individuals, clinical site was most significantly associated with host genetic profile (*R*^2^ = 0.009, *P* = 0.001), followed by microbiome PC1 (*R*^2^ = 0.008, *P* = 0.001) and disease status (COPD/healthy, *R*^2^ = 0.008, *P* = 0.032, Fig. S4). Microbiome PC1 was ranked the third and second in association with host genetics in COPD and healthy individuals, respectively. These results further support the close association between the airway microbiome and host genetic variation.

### Host genetic variations are associated with COPD microbiome features

Given the association between host genetic backgrounds and the airway microbial diversity, we sought to assess the relationships between individual SNPs and the airway microbiome features in COPD individuals. We chose to focus on a subset of microbiome species and functional modules that were reasonably abundant and important in the disease context (significantly different between COPD and controls). For each of the selected microbiome species and Kyoto Encyclopedia of Genes and Genomes (KEGG) functional modules (a total of 158 microbiome features, including 52 species and 106 KEGG modules, see Methods), we performed an association analysis with the concurrent host genetic variants using a general linear mixed model. A total of 12,198 candidate SNPs were identified with associations with all 158 microbiome features (*P* < 1.0 × 10^−5^, as suggestive significance), involving 3188 loci mapped to 2131 genes (Table S3). Of them, 276 SNPs involving 45 loci mapped to 30 genes reached genome-wide statistical significance (*P* < 5.0 × 10^−8^, Table S3). Of the 2131 genes, 113 genes were known candidate genes reported in previous GWAS studies in association with lung function or COPD (*P* < 5.0 × 10^−8^, Table S4), and were selected for further investigation to facilitate interpretation. The SNPs from these genes collectively exhibited 171 associations with 29 microbiome species and 48 KEGG modules (Table S4). Of them, the most significant association was found between *Streptococcus salivarius* and rs6917641 most proximal to *TBC1D32* (*P* = 9.54 × 10^−8^). This was followed by associations between *Xanthomonas euvesicatoria* and two SNPs, rs563696052 and rs368423146 in the intronic region of *ERC2* (*P* = 1.02 × 10^−7^) and *SLC1A2* (*P* = 1.60 × 10^−7^), respectively, and the association between *Moraxella catarrhalis* and rs74066259 in the intronic region of *SMIM2* (*P* = 2.28 × 10^−7^).

For KEGG modules, the strongest association was found between the assimilatory nitrate reduction (M00537) and rs7166844 in the intronic region of *SLC27A2* (*P* = 1.20 × 10^−7^), followed by associations involving ribose transport system (M00212, associated with rs6461680 mapped to *CARD11*), two modules related to secondary metabolism (M00418 and M00022, associated with rs7166844 mapped to *SLC27A2* and rs5803203 to *ENOX1*, respectively), and two modules related to bacterial two-component system (M00511) and transport system (M00592), respectively. Among host genes, *SLC27A2* exhibited significant associations with the greatest number of 6 microbiome features, followed by *ENOX1* associated with 4 microbiome features. Genetic association analysis using an expanded set of 517 species (relative abundance> 0.0001) and all 461 functional modules revealed a total of 164,236 SNPs associated with 6126 genes, among which 486 genes were candidate genes previously reported as genetically associated with COPD (*P* < 5.0 × 10^−8^, Table S5). Of them, 402 associations between 269 microbiome species, 55 functional modules, and 267 host genes reached genome-wide significance (*P* < 5.0 × 10^−8^, Table S6).

We also assessed association between airway microbiome and SNPs previously found to be associated with lung function or COPD with genome-wide significance (*P* < 5.0 × 10^−8^). In this regard, we have comprehensively searched public literatures and databases (GWAS catalog, Open Targets Genetics, GWAS Atlas) regarding GWAS studies for COPD and lung function. A total of 1427 SNPs from 19 datasets in GWAS catalog database derived from 10 studies associated with lung function measurements using genome-wide significance *P*-value 5 × 10^−8^ as cutoff were incorporated for further analysis [5, 29–37] (Table S7). We then associated these SNPs with microbiome taxa and functional modules in our dataset. Among the selected 52 microbiome taxa and 106 functional modules, 4 associations were identified involving 3 SNPs (mapped to *FOLH1B*, *WDR18* and *TMEM163*) and 4 microbiome features (*P* < 1.0 × 10^−5^, Table S7). Among all 517 microbiome taxa (identified using relative abundance cutoff 0.0001) and 461 functional modules, 50 associations were identified involving 21 SNPs and 40 microbiome features (*P* < 1 × 10^−5^, Table S7). Four associations involving SNP rs11666499 mapped to *LIMASI* and *Acinetobacter* species reached genome-wide significance (*P* < 5 × 10^−8^, Table S7).

*Lactobacillus salivarius* and *Lactobacillus oris* were identified as potential beneficial microorganisms mechanistically involved in COPD in our previous study based on the same cohort [13]. For *Lactobacillus salivarius*, a total of 76 SNPs involving 14 loci mapped to 11 genes were identified (Table S8), with rs7913363 (mapped to *BBP1*, *PDCD4*, and *SHOC2*) being the most significant (*P* = 2.57 × 10^−7^). For *Lactobacillus oris*, a total of 168 SNPs involving 17 loci mapped to 9 genes were identified (Table S8), with rs6996846 (mapped to *AF131215.5* and *AKSMO*) being the most significant (*P* = 2.24 × 10^−6^). None of these genes, however, were previously reported to be genetically associated with COPD.

Among SNPs associated with lung function, none were found to be significantly associated with microbiome features in our dataset (Tables S2, S4). In addition, none of these SNPs or genes associated with inflammatory endotype or clinical traits overlapped with those associated with the microbiome features (Tables S2, S4). On the other hand, at the transcriptomic level, among all 113 host genes involved in microbiome-host genetic associations, 46 genes were found to be associated with sputum neutrophil, 3 genes associated with sputum eosinophil, 25 genes associated with GOLD status, 8 genes associated with ICS, and 1 gene associated with smoking (FDR < 0.05, Table S4). These results suggest a possibly greater impact of these clinical factors on the microbiome-host gene associations at the transcriptomic than the genetic level. Collectively, these results suggest the genetic associations between COPD-associated human genes and the airway microbiome taxonomic and functional features that could imply possible microbiome-host interactions.

### The airway microbiome-host genetic associations were transcriptionally linked

**Fig. 3:**
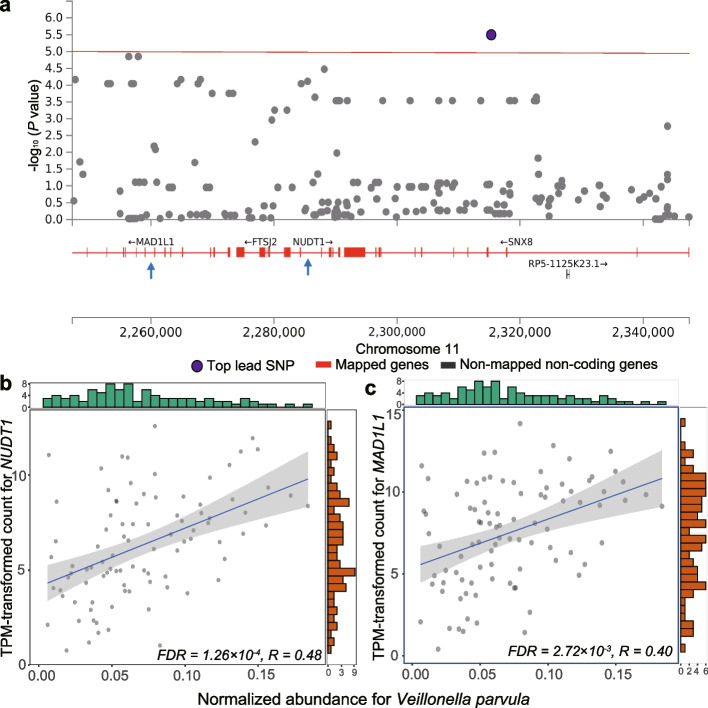
The correlation between the airway microbiome taxa and its genetically associated host genes at the transcriptomic level in COPD patients. **a** Regional manhattan plot showing the associations between *Veillonella parvula* and the host genetic variants in *MAD1L1* and *NUDT1*. The mapped genes are marked in red and the top lead SNP is colored in purple. The red line represents the *P*-value of 1.0 × 10^−5^. **b**, **c** Scatterplots for the significant correlation between the normalized abundance of *Veillonella parvula* (x-axis) and the expression of *NUDT1* and *MAD1L1* (y-axis) in COPD patients. The green and red bars are histograms showing the distribution of the X and Y axis

**Fig. 4:**
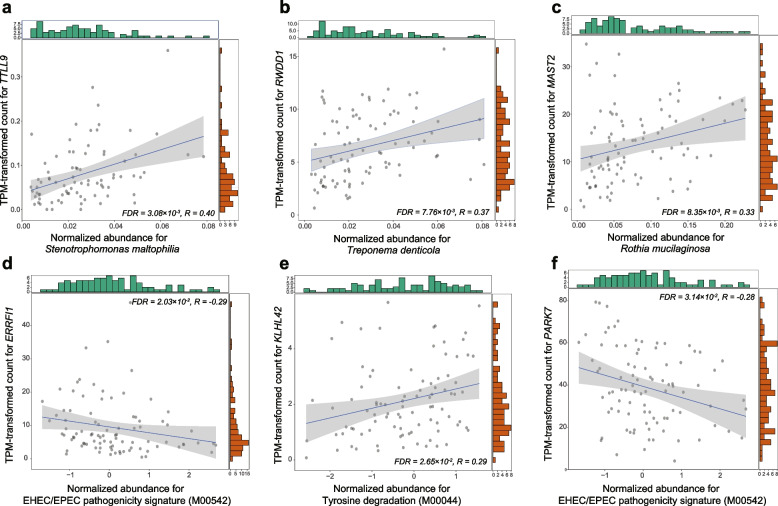
Additional correlations between the airway microbiome features and their genetically associated host genes at the transcriptomic level in COPD patients. **a-c** Scatterplots for the significant correlations of three microbiome species-level taxa *Stenotrophomonas maltophilia*, *Treponema denticola*, *Rothia mucilaginosa* with the expression of *TTLL9*, *RWDD1*, and *MAST2*, respectively, in COPD patients. **d-f** Significant correlations of two microbiome functional modules tyrosine degradation (M00044), and EHEC/EPEC pathogenicity signature (M00542), with the expression of *KLHL42*, *ERRFI1* and *PARK7*. The green and red bars are histograms showing the distribution of the X and Y axis

We next analyzed concurrent airway host transcriptomic data to assess any genetically associated microbiome features and host genes that were further correlated at the transcriptional level. Among the 122 microbiome-host genetic associations, 27 significant correlations were identified between the transcriptional level of 24 host genes and 12 microbiome species and 10 KEGG modules (Spearman correlation, *P* < 0.05, Table 1). The most significant correlations were found between *Veillonella parvula* and *NUDT1* (rho = 0.48, FDR = 1.26 × 10^−4^) and *MAD1L1* (rho = 0.40, FDR = 2.72 × 10^−3^), both proximal to rs62442525 that exhibited genetic association with *V. parvula* (Fig. 3a-c). This was followed by correlations of *Stenotrophomonas maltophilia*-*TTLL9* (Fig. 4a, Fig. S5**,** rho = 0.40, FDR = 3.08 × 10^−3^, rs9967912), *Treponema denticola-RWDD1* (Fig. 4b, rho = 0.37, FDR = 7.76 × 10^−3^, rs6568956), *Rothia mucilaginosa*-*MAST2* (Fig. 4c, rho = 0.33, FDR = 8.35 × 10^−3^, rs79257400), *Prevotella intermedia*-*BACR1* (rho = 0.34, FDR = 5.73 × 10^−2^, rs7204848), and *Haemophilus influenzae*-*LTA4H* (rho = 0.30, FDR = 6.21 × 10^−2^, rs56396137). Of note, a non-significant positive correlation was also found between *S. salivarius* and *TBC1D32* (rho = 0.23, FDR = 8.60 × 10^−1^, rs6917641, Table 1**,** Table S4) that exhibited the strongest genetic association. Among microbial functional modules, the most significant correlation was between *ERRFI1* and EHEC/EPEC pathogenicity signature (Fig. 4d, Fig. S5, M00542, rho = − 0.29, FDR = 2.03 × 10^−2^), followed by *KLHL42* and microbial tyrosine degradation (Fig. 4e, M00044, rho = 0.29, FDR = 2.65 × 10^−2^), and *PARK7* and EHEC/EPEC pathogenicity signature (Fig. 4f, M00542, rho = − 0.28, FDR = 1.95 × 10^−2^). These results suggest that the airway microbiome-host genetic associations can be further correlated at the transcriptional level, providing a possible explanation for host genetic variations influencing the microbiome.

**Table 1:** Correlations between the host gene expression and the genetically associated airway microbiome taxa and modules in COPD patients

Microbiome taxa and modules	SNP	Chr	Major allele	Minor allele	*P*-value	Gene	Spearman *P*-value	FDR	Rho
*Veillonella parvula*	rs62442525	7	G	A	3.20E-06	*NUDT1*	1.11E-06	1.26E-04*	0.483
*Veillonella parvula*	rs62442525	7	G	A	3.20E-06	*MAD1L1*	7.59E-05	2.72E-03*	0.401
*Stenotrophomonas maltophilia*	rs9967912	20	A	C	4.00E-06	*TTLL9*	1.80E-04	3.08E-03*	0.402
*Treponema denticola*	rs6568956	6	A	C	5.46E-06	*RWDD1*	3.67E-04	7.76E-03*	0.371
*Rothia mucilaginosa*	rs79257400	1	C	G	1.90E-06	*MAST2*	1.52E-03	8.35E-03*	0.331
*Prevotella intermedia*	rs7204848	16	A	G	9.14E-07	*BCAR1*	1.17E-03	5.73E-02	0.335
*Haemophilus influenzae*	rs56396137	12	A	G	2.09E-06	*LTA4H*	6.76E-03	6.21E-02	0.304
*Haemophilus influenzae*	rs4352337	3	G	A	5.03E-06	*KBTBD12*	8.91E-03	7.20E-02	0.294
*Campylobacter concisus*	rs4683647	3	C	T	6.00E-06	*ATP1B3*	1.11E-02	7.84E-01	0.265
*Neisseria mucosa*	rs6557999	8	A	G	8.85E-06	*PTK2B*	1.99E-02	8.05E-01	−0.246
*Streptococcus salivarius*	rs6917641	6	A	G	9.54E-08	*TBC1D32*	3.57E-02	8.60E-01	0.228
*Streptococcus pneumoniae*	rs147224807	5	T	G	4.13E-06	*AFF4*	2.95E-02	8.89E-01	−0.242
*Treponema denticola*	rs6568956	6	A	C	5.46E-06	*RSPH4A*	3.91E-02	4.75E-01	0.220
*Xanthomonas euvesicatoria*	rs111818593	12	A	G	2.54E-06	*MRPS35*	3.13E-02	3.84E-01	−0.234
*Ralstonia insidiosa*	rs34568075	6	A	–	3.70E-06	*TBC1D32*	3.82E-02	9.14E-01	0.224
M00542: EHEC/EPEC pathogenicity signature	rs34823376	1	G	A	4.70E-06	*ERRFI1*	5.75E-03	2.03E-02*	−0.289
M00044: Tyrosine degradation	rs12311799	12	G	A	8.18E-06	*KLHL42*	5.17E-03	2.65E-02*	0.289
M00542: EHEC/EPEC pathogenicity signature	rs34823376	1	G	A	4.70E-06	*PARK7*	8.25E-03	3.14E-02*	−0.277
M00375: Hydroxypropionate-hydroxybutylate cycle	rs150193199	11	G	C	9.19E-06	*NARS2*	3.08E-03	4.67E-02*	−0.317
M00338: Cysteine biosynthesis	rs545977123	11	G	C	1.38E-06	*EPS8L2*	7.78E-03	5.54E-02	−0.28
M00332: Type III secretion system	rs34823376	1	G	A	6.20E-06	*ERRFI1*	3.14E-03	5.98E-02	−0.31
M00218: Fructose transport system	rs17121175	1	C	A	9.00E-06	*NFIA*	2.56E-02	9.02E-02	−0.234
M00332: Type III secretion system	rs34823376	1	G	A	6.20E-06	*PARK7*	6.98E-03	9.86E-02	−0.284
M00512: CckA-CtrA/CpdR (cell cycle control) two-component regulatory system	rs17824678	12	G	A	4.63E-06	*MSRB3*	2.30E-02	1.19E-01	0.244
P02010: ABC transporters	rs146351733	11	A	–	4.04E-06	*PDGFD*	4.00E-02	1.63E-01	−0.215
M00540: Benzoate degradation	rs1741618	20	T	A	4.35E-06	*EEF1A2*	4.33E-02	3.71E-01	−0.215
M00511: PleC-PleD (cell fate control) two-component regulatory system	rs5746415	22	A	G	2.86E-07	*CECR2*	2.78E-02	5.10E-01	−0.236

*MR* Mendelian randomization, *IVW* inverse variance weighted, *beta* beta coefficient, *se* standard error, *OR* Odds ratio, *CI* Confidence interval

*FDR-adjusted *P*-value< 0.05

**Fig. 5:**
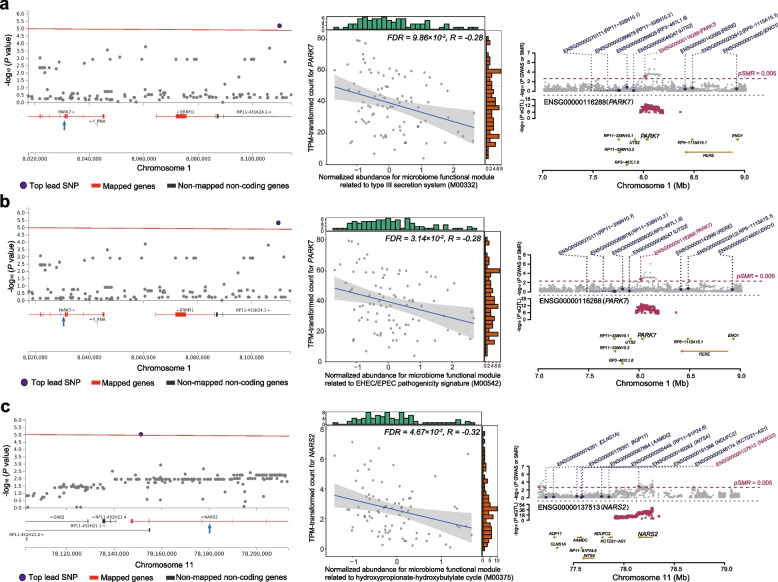
The correlation between the airway microbiome functional modules and its genetically associated host genes at the transcriptomic level in COPD patients. Regional manhattan plots on the left show the associations between three KEGG modules (M00332, M00542, M00375, **a-c** with the host genetic variants in *PARK7 and NARS2*. The mapped genes are marked in red and the top lead SNP is colored in purple. The red line represents the *P*-value of 1.0 × 10^−5^. Scatterplots in the middle show the significant correlation of the three KEGG modules (x-axis) and the expression of host genes *PARK7 and NARS2* (y-axis). The plots on the right show the corresponding genetic loci from SMR analysis. The gray dots in the top mannattan plots show *P*-values for SNPs associated with KEGG modules. The bottom plots represent the eQTL *P*-values of SNPs from the GTEx study for probe ENSG00000116288 tagging *PARK7* and ENSG00000137513 tagging *NARS2*. The genes (*PARK7 and NARS2*) that passed SMR and HEIDI tests are highlighted in red

To further explore any potential causal associations between the host gene expression and microbiome features, we integrated the lung gene expression quantitative trait loci (eQTL) and gene splicing quantitative trait loci (sQTL) data from Genotype-Tissue Expression (GTEx) database, and performed a summary-data-based Mendelian randomization (SMR) analysis using the summary data sets of the above significant microbiome-host gene pairs, with host genes as exposure and microbiome features as outcome. The eQTL analysis revealed significant associations between microbial type III secretion system (M00332), enteropathogenic and enterohemorrhagic *Escherichia coli* (EHEC/EPEC) pathogenicity signature (M00542) and *PARK7* (Fig. 5a-b, Fig. S6, *P*_*SMR*_ = 1.72 × 10^−3^ and *P*_*SMR*_ = 1.63 × 10^−3^, Table S9), and between hydroxypropionate-hydroxybutylate cycle (M00375) and *NARS2* (Fig. 5c, Fig. S6, *P*_*SMR*_ = 2.90 × 10^−3^, Table S9). The sQTL analysis identified significant associations between *PARK7* and M00332 (Fig. S7, *P*_*SMR*_ = 3.71 × 10^−3^ and *P*_*SMR*_ = 3.69 × 10^−3^) and M00542 (*P*_*SMR*_ = 3.01 × 10^−3^ and *P*_*SMR*_ = 2.99 × 10^−3^, Table S9). These results support the possibility that host genetic variation could influence the airway microbial functions through transcription or splicing activities.

### Mendelian randomization revealed potential microbiome-COPD associations

To further explore any genetically mediated causal relationships between COPD and the airway microbiome, we performed a two-sample bidirectional Mendelian randomization (MR) analysis. We selected 13 qualified SNPs as instrumental variables (IVs) (*P* < 5.0 × 10^−8^, *r*^2^ < 0.02, clumping window = 5000 kb) from the study of Ishigaki et al. that includes an eastern Asian population of 3315 cases and 201,592 controls [38]. Eleven of these 13 qualified SNPs were implemented in our MR analysis after extracting the IVs from outcome GWAS summary data and removing the palindromic SNPs. The *F-*statistics of IVs are all greater than 10 (range: 31.3–71.2), indicating no evidence of instrument bias (Table S10). MR analyses were performed using COPD as exposure and the 158 microbiome features (52 species and 106 modules) as outcomes.

**Fig. 6:**
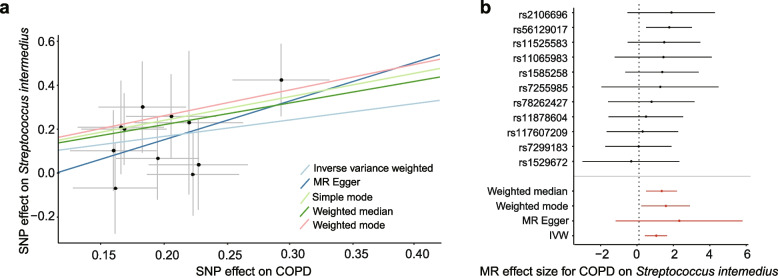
Mendelian randomization for a potential causal relationship between COPD and *Streptococcus intermedius*. **a** The scatterplot showing the SNP effects on COPD versus the relative abundance of *S. intermedius*, with the slope of each line corresponding to the estimated MR effect using each method. **b** Forest plot showing the MR-estimated effect sizes for COPD on *S. intermedius* for individual SNPs and their combinations

A causal association was found between COPD and increased relative abundance of *Streptococcus intermedius* (Fig. 6a, Table 2, OR (95 CI): 2.53 (1.38, 4.70); *P* = 0.0028) using the inverse-variance weighted (IVW) method. This finding was also supported by the weighted median and weighted mode methods (Table 2, OR (95 CI): 3.42 (1.54, 7.64); *P* = 0.0029; OR (95% CI): 4.31 (1.17, 15.83); *P* = 0.048). Forest plots of causal effects using a single SNP showed that none of them was extremely significant for association between exposure and outcome (Fig. 6b). The leave-one-out sensitivity analysis demonstrated that the associations were not driven by any specific SNPs (Fig. S8). No horizontal pleiotropy and no heterogeneity was found between the individual SNPs (Table S11). When analyzing in the opposite direction, no association was found between COPD and airway microbiome features using either of the MR methods (Table 2). These findings suggest a potential causal association between COPD and increased airway *S. intermedius* mediated through host genetics.

**Table 2:** Bidirectional MR results for the relationship between COPD and the relative abundance of *Streptococcus intermedius*

Directionality	MR methods	Number of SNPs	F-statistic	beta	se	OR (95% CI)	*P*-value
Effect of COPD on *S. intermedius*	IVW	11	42	0.93	0.31	2.53 (1.38,4.70)	0.0028
	Weighted median	11	42	1.23	0.41	3.42 (1.54,7.64)	0.0029
	Weighted mode	11	42	1.46	0.65	4.31 (1.17,15.83)	0.048
	Simple mode	11	42	1.34	0.73	3.82 (0.90,16.30)	0.095
	MR-Egger	11	42	2.20	1.77	9.03 (0.28,294.23)	0.25
Effect on *S. intermedius* on COPD	IVW	14	31	0.0076	0.01	1.00 (0.99,1.03)	0.42
	Weighted median	14	31	0.013	0.013	1.01 (0.99,1.04)	0.33
	Weighted mode	14	31	0.015	0.02	1.02 (0.98,1.06)	0.46
	Simple mode	14	31	0.016	0.021	1.02 (0.97,1.06)	0.47
	MR-Egger	14	31	0.021	0.038	1.02 (0.95,1.10)	0.59

*MR* Mendelian randomization, *IVW* Inverse variance weighted, *beta* Beta coefficient, *se* Standard error, *OR* Odds ratio, *CI* Confidence interval

## Discussion

Here, through a microbiome-host co-profiling based on the airway metagenomic sequencing data, we reported the associations between the airway microbiome and host genetic polymorphisms in COPD. We acquired over 5 million high-quality SNPs and validated some of the SNPs in relation to genes previously reported as associated with lung function, demonstrating the possibility in obtaining biologically interpretable host genetic information from airway metagenomic data [39]. Importantly, host genetic variation exhibited a greater association with the airway microbiome than all other clinical and demographic factors that we have surveyed, except for geography and disease status, suggesting that it could be a critical but overlooked intrinsic factor shaping the airway microbiome.

We identified genetic associations between human genes and specific airway microbial taxonomic and functional features in COPD individuals. Among all candidate genes, the strongest association was found between *S. salivarius* and rs6917641 located in the intronic region of *TBC1D32*, a gene previously reported to be genetically associated with a broad range of respiratory disorders including emphysema, asthma, and rhinitis [5, 40, 41]. *S. salivarius* was found to be increased in COPD in particular in GOLD I patients [42] and was recently reported to have a multi-functional role in promoting inflammation leading to allergic rhinitis [43] and inducing experimental pulmonary hypertension [44]. Of note, the same host gene was also genetically associated with *Ralstonia insidiosa* (*P* = 3.7 × 10^−6^), an opportunistic lung pathogen [45]. *TBC1D32* was involved in ciliary function and Sonic hedgehog signaling [46, 47], both implicated in COPD pathogenesis. Genetic alteration of this gene might therefore lead to cilia and epithelial dysfunction and inflammation in COPD and broader airway diseases through elevation of pathogenic members of the airway microbiota. From the host perspective, *SLC27A2*, a long-chain fatty acid transporter that is involved in host neutrophil degranulation [48], was found to be genetically associated with a diverse microbial metabolic functionality, including nitrate and sulfate reduction, toluene degradation, methanogenesis, and inositol transport, implying a possible role of this gene interacting with the microbial metabolisms through neutrophil activities.

Through integrating host transcriptomics, we further identified multiple genetically associated host genes and microbiome features that were correlated at the transcriptomic level. Notably, these microbiome-host gene associations were both supported at the genetic and transcriptomic level, implying that genetic variation of the host genes may potential influence the airway microbiome through their expression activities. For instance, *Veillonella parvula,* which was found to activate airway inflammation and impair the bronchial epithelial activities [49, 50], was genetically linked and transcriptionally correlated with *NUDT1* and *MAD1L1*. Given the function of *MAD1L1* in reducing telomerase activity, the co-altered microbial-host features could synergistically result in the reduced proliferation of epithelial cells and contribute to emphysema [51]. In addition, *Haemophilus influenzae*, a key player in COPD airway microbiota, was found to be genetically and transcriptionally associated with *LTA4H* encoding leukotriene A4 hydrolase that can be converted to neutrophil attractant leukotriene B4 in promoting emphysema [52]. This provides a plausible explanation on persistence of neutrophilic inflammation in COPD individuals colonized with *H. influenzae* [53, 54]*. Stenotrophomonas maltophila*, an airway pathogen associated with COPD and other chronic airway diseases, was found to be associated with *TTLL9*, a tubulin tyrosine ligase gene important for airway epithelial cilia function [55]. It is possible that genetic alteration of this gene may lead to airway cilia dysfunction leading to susceptibility of infection of this pathogenic bacterium. Likewise, *RSPH4A*, another gene involved in airway cilia function [56], was associated with *Treponema denticola*, an oral bacterium with a pro-inflammatory role [57]. On the other hand, *Rothia mucilaginosa*, an airway bacterium recently reported to have an anti-inflammatory role [58], was associated with *MAST2* encoding microtubule-associated serine/threonine kinase whose dysfunction is involved in pulmonary vascular remodeling [59], together indicating a possible link between airway vascular structural changes, inflammation and microbial dysbiosis in COPD.

Through SMR analysis, we further observed a potential causal link between the expression of *PARK7* and two metagenomic functional modules in type III secretion system essential for bacterial virulence. *PARK7* is an antioxidant gene that acts as a stabilizer of the transcription factor Nrf2 to facilitate its effects [60]. The deficiency of *PARK7* was found to impact the gut microbiota [61] and impair bacterial clearance in sepsis [62], suggesting it may have a role in shaping the homeostasis of the microbial community. Genetic alteration may lead to decreased expression of *PARK7* and elevation of bacterial virulence potentials, which could interact with each other in together promoting COPD oxidative stress [63]. Through these integrative analyses, we were able to step-by-step refine the hypotheses for the interaction between the airway microbiome and COPD host genetic variations.

To further explore potential causality between the airway microbiome and host genetic variations, we performed a bidirectional MR analysis between the microbiome features and COPD. We found an increase in the relative abundance of airway *Streptococcus intermedius* that could be determined genetically in COPD. *S. intermedius* is a part of the *Streptococcus milleri* group and is considered as a commensal member of the airway microbiota, while it can also act as an opportunistic pathogen causing purulent infection and abscess in the lung [64]. COPD has been reported as a risk factor for *S. intermedius* overgrowth [65], while the elevation of *S. intermedius* can in turn lead to COPD exacerbations [66]. In light of our findings, it is plausible that *S. intermedius* may proliferate in response to the altered lung microenvironment in COPD and contribute to the self-perpetuating COPD-dysbiosis cycle that predisposes to exacerbations and disease progression [67].

Despite novel relationships identified between the airway microbiome and COPD host genetics, several important limitations are noted. First, the participants are of eastern Asian ancestry and, as a pilot study, the cohort size is small. Therefore, the results can only be viewed as hypothesis-generating that remain to be validated in larger populations with different genetic backgrounds and ethnicities, using other conventional genotyping approaches such as SNP arrays, and through in vivo and in vitro experiments. Compared with American and European populations, genetic investigations on Asian COPD populations remain under-represented, which could have affected the performance of genotyping and imputation accuracy [68]. Second, despite a deep metagenomic sequencing, the coverage of the human genome remains moderate (~10x), which could have affected the SNP calling performance [69]. Third, a comprehensive assessment of environmental factors for the COPD individuals such as indoor (i.e. biofuel use, occupational pollution) and outdoor (PM_2.5_ concentration) pollution is lacking, which could have led to an overestimation on the relative importance of host genetics on the microbiome. Fourth, due to heterogeneity of COPD, inflammatory endotype, severity, and other clinical factors such as ICS usage and smoking could potentially impact our results, as they were associated with the transcriptional level of genes involved in microbiome-host genetic association. Due to the small sample size, it is currently impossible to subdivide COPD individuals according to these factors and perform microbiome-host genetic sub-analysis. Further larger-scale studies are warranted to more explicitly assess the impact of clinical factors on microbiome-host genetic associations. Fifth, principal component analysis (PCA) was conducted to assess the overall genetic variability and its associations with microbiome alpha and beta diversity. There are limitations in the application of PCA to genetics data, including the modest proportion of variation it explains and the lack of biological interpretability for the PCs. Although imperfect, PCA remains a reasonable approach applied in existing microbiome-genetic studies [70–73]. And by using a ‘bi-directional’ PERMANOVA, our results further support the close association between the airway microbiome and host genetics. Last, despite the implementation of MR analysis which was designed for causal inferences, the precise causality between microbiome and host genetics cannot be established and warrants further investigations through experimental and mechanistic studies.

In summary, our study demonstrates the feasibility in uncovering host genetic associations of the airway microbiome through microbiome-host co-profiling using deeply sequenced metagenomics. Results of this study suggest a previously underappreciated role of host genetics in shaping the airway microbiome and provide fresh hypotheses for host genetic-microbiome interactions that could contribute to COPD pathogenesis.

## Methods

### Patients and samples

The characterization of the metagenomes (with proper reagent controls) and host transcriptomes for this cohort has been described previously [13]. Briefly, induced sputum samples were collected from patients with stable COPD (*n* = 72) and age-matched healthy controls (*n* = 18) in the First Affiliated Hospital of Guangzhou Medical University, Guangzhou, China, and patients with stable COPD (*n* = 27) and healthy controls (*n* = 18) in Shenzhen People’s Hospital in Shenzhen, China, respectively. For Shenzhen cohort, the two groups are generally age-matched, with healthy controls being non-significantly younger than the COPD individuals (*P* = 0.061). All COPD patients met the diagnostic criteria according to GOLD [74]. All 135 individuals were subject to deep sputum metagenomic sequencing, and 130 sputum samples were subject to concurrent host transcriptomic profiling. For COPD patients, the inclusion criteria were: (1) age > 40 years; and (2) confirmed diagnosis of COPD according to the GOLD guideline (post-bronchodilator forced expiratory volume in 1 s [FEV_1_]/forced vital capacity [FVC] ratio < 0.7). The exclusion criteria were: (1) physician-diagnosis of asthma or significant respiratory disease other than COPD; (2) COPD exacerbation within 4 weeks of enrollment; (3) history of lung surgery and tuberculosis; (4) diagnosis of cancer; (5) blood transfusion within 4 weeks of enrollment; (6) diagnosis of autoimmune diseases; (7) enrollment in a blinded drug trial; and (8) short-term antibiotic usage within 4 weeks of enrollment. Informed consent was obtained from all patients. This study was approved by the ethics committee of the two centers (reference no. 2017–22 and KY-LL-2020294-01). All participants provided informed consent. All raw sequencing data were deposited for strictly controlled access only (see data and code availability), to protect the privacy of the donor genotyping information which is highly confidential.

### Quality control of sputum samples

Quality control of the sputum was performed upon collection. Specifically, sputum plugs were separated from saliva, and were diluted with 0.1% dithiothreitol solution and filtered through a 48 μm nylon-mesh filter following a standardized sputum processing protocol [75, 76]. The numbers of total cells, leucocytes, and squamous epithelial cells were recorded. Sputum specimens with a squamous epithelial cell to leucocyte ratio < 1:2.5 were regarded as with minimal contaminations from oropharyngeal materials and eligible for subsequent experiments [77].

### Multi-omic sequencing and analyses

DNA was extracted from quality-controlled sputum plugs using the Qiagen DNA Mini kit and was deep-sequenced using Illumina NovaSeq platform (2 × 150 bp, targeted ≥30G sequences per sample). Four reagent controls (DNA extraction blanks) were used for sequencing, two for Guangzhou and two for Shenzhen cohorts respectively. The results for the reagent controls were described previously [13]. Bacterial species identified in at least two of four reagent controls with relative abundance greater than 0.001 were excluded from downstream analyses. The cell and supernatant isolation was performed on the remaining sputum using a two-step method with a PBS wash step followed by a dithiothreitol step and cytospins according to a standardized sputum processing protocol [75, 76]. RNA was extracted from sputum cells using the Qiagen RNase Mini kit for mRNA-sequencing using Illumina NovaSeq platform (2 × 150 bp).

The characterization of the airway metagenomes and the host transcriptomes was described previously [13]. Briefly, raw metagenomic reads were processed using the Sunbeam pipeline [78], in which quality control was performed using Cutadapt (v.2.5) [79], reads were filtered using Komplexity [78], and host reads were filtered by mapping to human genome GRCh38 using BWA(v.0.7.17) [80]. For both the actual sputum samples and negative reagent controls, taxonomic profiling was performed using Kraken 2 (v.2.0.8) [81]. Bacterial species with relative abundance> 0.001 and identified in at least two of four reagent controls were regarded as potential contaminants and filtered out from subsequent analyses, as described previously [13]. A total of 33 species-level taxa were removed during this step (Table S12), constituting an average of 0.019% of the abundance of all taxa from the actual samples [13]. For host transcriptome, raw reads were quality-filtered using Cutadapt (v.2.5) [79] and aligned to the human genome GRCh38 using Hisat2 (v.2.1.0) [82]. RSEM (v.1.3.3) [83] was used to generate the gene expression count table.

### WGS alignment and SNP calling

The human host reads from the metagenomic data were subject to genotyping. Specifically, the Genome Analysis Toolkit’s (GATK v4.1) [84] BaseRecalibrator was used to create recalibration tables and to screen for known SNPs in the BAM files from dbSNP (v138). Base Quality Score Recalibration (BQSR) was used for subsequent base quality recalibration and removal of read pairs with improperly aligned segments. The genetic variants were generated using GATK’s HaplotypeCaller. GVCFs containing SNPs created from HaplotypeCaller were then combined (CombineGVCFs), genotyped (GenotypeGVCFs), selected (SelectVariants), variant score recalibrated (VariantRecalibrator), and filtered (ApplyVQSR) in GATK. For the GATK VariantRecalibrator process, we used our variants as inputs and four standard SNP sets to train the model: (1) dbSNP builds 138 SNPs; (2) 1000 Genome phase 3 high confidence SNPs; (3) 1000 Genomes Project SNPs from Omni 2.5 chip; (4) HapMap3.3 SNPs. The sensitivity threshold was set as 99.9% for SNPs for the variant selection. A set of 26,165,582 raw SNPs were obtained after applying the filtration.

### Quality control and imputation

The below inclusion thresholds were applied for quality control of the variants using PLINK (v.2.0) [85]: (1) genotype calling rate > 95%; (2) minor allele frequency (MAF) > 0.05; (3) Hardy-Weinberg equilibrium (HWE) *P* > 0.0001 and finally obtain 1,082,482 high-quality variants. The genomic coordinates were converted from GRCh38 to GRCh37 using Crossmap (v.0.5.4) and the high-quality variants were imputed using BEAGLE (v.5.2) [86], with the 1000 Genomes phase 3 Project as a reference panel (ne = 20,000, window = 100, seed = − 99,999). The variants with imputation information> 0.7 were retained and further filtered to keep variants with MAF > 0.05 using PLINK (v.2.0) [85]. Ultimately, a set of 5,471,650 high-quality SNPs were retained. Two COPD individuals were identified as notable outliers in the principal coordinate analysis based on SNP dosage information and excluded from downstream analyses. The SNP missing rate of these two samples were significantly higher than all other samples (14.85% for Z031 and 10.97% for K013V0, compared with 1.2% ± 1.2 for the remaining samples), which could be partly due to their lower ratio of reads passing quality control (61.9 and 76.3%, compared with 83.9% ± 7.3% for the remaining samples) and reads mapping to the human genome (35.4 and 46.8%, compared with 77.3% ± 12.0% for the remaining samples).

### Functional mapping and annotation

Gene annotation was performed using SNP2GENE in FUMAGWAS (http://fuma.ctglab.nl/) [87]. The *P*-value threshold was set with 1.0 × 10^−5^, r^2^ threshold to define independent significant SNPs and lead SNPs were set as 0.6 and 0.1 respectively. The genetic data of East Asian populations in 1000G phase 3 were viewed as reference data to conduct LD analyses. The maximum distance between LD blocks to merge into a genomic locus was 250 kb. We used the positional mapping method and maps variants to genes based on physical distance within a 10 kb window [87] The mapped genes were further annotated in databases including Open Targets Genetics (https://genetics.opentargets.org/), GWAS Catalog (https://www.ebi.ac.uk/gwas/), and GWASATLAS (https://atlas.ctglab.nl/) [88–90] to assess whether the genes were reported to be genetically associated with COPD in previous studies.

### Association between host genetics, lung function and the microbiome

Genome wide association studies (GWAS) of lung function measurement (FEV_1_/FVC) was performed in COPD individuals only (*n* = 97) and in all individuals (*n* = 133) using a general mixed linear model, adjusting for site, age, sex, BMI, smoking status, the top 10 principal components (PCs) [91] of the genotype data generated by using SNP dosage in PLINK (v.2.0) [85] and kinship matrix between individuals generated in GEMMA (v.0.98.5) [92]. The *P*-values of the genome-wide association were adjusted using the wald test in GEMMA (v.0.98.5) [92].

The associations between the first PC of the host genetic variations and the microbiome beta diversity (based on Bray-Curtis distance at the species level) were assessed with permutational multivariate analysis of variance (PERMANOVA) [93] in all samples (*n* = 133) and within COPD (*n* = 97) and control (*n* = 36) group respectively using vegan [94] in R (v.4.2.0). Correlations between the first PC of host genetic data and first PC and richness of the airway microbiome data were performed with spearman correlation in R (v.4.2.0) in all the samples (*n* = 133). The associations between microbiome taxonomic and functional features and host SNPs were assessed using a general mixed linear model with adjustment for site, age, sex, BMI, smoking status, the top 10 principal components (PCs) [91] of the genotype data, and kinship matrix between individuals in GEMMA (v.0.98.5) [92].

For taxonomic features, the microbiome species-level taxa enriched in COPD (FDR < 0.05, *n* = 6) or in healthy individuals (*n* = 25) as well as those with average relative abundance> 0.005 in COPD (*n* = 21) were selected, resulting in a total of 52 species. For functional features, the KEGG modules significantly enriched in COPD (FDR < 0.05, *n* = 70) and health (*n* = 36) were selected, resulting in a total of 106 modules. Together, these resulted in a total of 158 microbiome features for downstream analyses. The taxonomic relative abundances were arcsin square root transformed followed by z-score normalization and the abundances of the KEGG modules were z-score normalized. The *P*-values of the genome-wide association were adjusted by wald test in GEMMA (v.0.98.5) [92]. Host gene expression count was TPM-transformed. Spearman correlations were investigated between TPM values of host genes and normalized abundances of the microbiome features. Regional Manhattan plots were made in FUMAGWAS (http://fuma.ctglab.nl/) [87].

### Summary-based Mendelian randomization (SMR) analysis

SMR analysis was performed to identify the expression and the alternative splicing level of host genes associated with the airway microbiome features in COPD [99]. Lung expression quantitative trait loci (eQTL) summary statistics were obtained from the GTEx eQTL summary dataset (version 8) [95]. As genetic variations may function as a regulator of gene-splicing events, the lung-splicing quantitative trait loci (sQTL) summary statistics (GTEx version 8) were also implemented in the SMR analysis. The statistical significance level was set to *P* < 0.0033 and *P* < 0.0042 respectively based on multiple testing corrections for the number of the SMR analysis (*n* = 15 for microbiome species-level SMR and *n* = 12 for module-level SMR, respectively). The heterogeneity in dependent instruments (HEIDI) test was conducted to assess heterogeneity in the association statistics by identifying the presence of any underlying single causal genetic variant from linkage in SMR analysis. The non-significant probes for heterogeneity (*P*_*HEIDI*_ ≥ 0.05) were retained [96].The SMR locus plots were generated by using the code in https://yanglab.westlake.edu.cn/software/smr/#SMRlocusplot19.

### Mendelian randomization (MR) analysis

A systematic two-sample MR analysis was applied to assess whether COPD can be potentially linked to the airway microbiome through host genetic variations [97]. Candidate instrumental variables (IVs) for COPD were selected at the *P* < 5 × 10^−8^ significance according to the study of Ishigaki et al. (2020) that includes 3315 cases and 201,592 controls of East Asian ancestry [98]. SNPs associated with COPD were clumped using extract_instruments in TwoSampleMR (v.0.3.4) [99] to retain only independent SNPs. The linkage disequilibrium (LD) threshold was set as *r*^2^ < 0.02 and a clumping window of 5000 kb [100, 101]. To assess the strength of the selected SNPs, the following equation was used to calculate the *F* statistics [102]:

$$F= PVE\times \left(N-2\right)/\left(1- PVE\right)$$

where N represents the effective GWAS sample size [103]. The PVE refers to the proportion of variance in phenotype explained by a given SNP [104]:

$$(PVE)=\left[2\ast \left( beta\hat{\phantom{0}} 2\right)\ast MAF\ast \left(1- MAF\right)\right]/\left[2\ast \left( beta\hat{\phantom{0}} 2\right)\ast MAF\left(1- MAF\right)+\left(\left( se(beta)\right)\hat{\phantom{0}} 2\right)\ast 2\ast N\ast MAF\ast \left(1- MAF\right)\right]$$

where N is the sample size, se is the standard error of effect size for the genetic variant of interest, beta represents effect size for the genetic variant of interest, and MAF is the minor allele frequency for the genetic variant of interest. An *F*-statistic≥10 indicates no strong evidence of weak instrument bias. For multiple IVs, we computed the mean *F* statistic across IVs [102, 103]. A total of 158 microbiome-GWAS summary data sets including those for the above mentioned 52 microbial species and 106 functional modules were used as outcomes. The inverse variance weighted (IVW) method was employed to estimate the effect size. Four additional MR methods were employed, including weighted median, MR-Egger, weighted mode, and simple mode. Three types of sensitivity analyses were performed, including the heterogeneity test, pleiotropy test, and leave-one-out analysis. The heterogeneity was quantified by Cochran’s Q statistic. The intercept of the MR-Egger regression test was performed to provide an estimate of the degree of directional pleiotropy [42]. The leave-one-out analysis was performed to evaluate whether the significant results were driven by a single SNP [105]. To explore whether the airway microbiome may causally impact COPD through host genetics, a reverse MR analysis was performed with airway microbiome as exposure and COPD as outcome. All analyses were conducted using TwoSampleMR (v.0.3.4) [99] in R (v.4.2.0). A STROBE-MR checklist was included in Table S13.

### Supplementary Information

**Supplementary Material 1.**

**Supplementary Material 2.**

## Data Availability

The raw metagenomic data for the human cohort were deposited in the Genome Sequence Archive in BIG Data Center (https://ngdc.cncb.ac.cn/), Beijing Institute of Genomics (BIG), Chinese Academy of Sciences, under PRJCA012278 for controlled access only, so that the privacy of the donor genotype information is strictly protected. The human sequence-depleted metagenomic data have been deposited in the European Genome-phenome Archive (EGA) under EGAS00001006398. The raw transcriptomic data for the human cohort have been deposited in the Chinese National Gene Bank Nucleotide Sequence Archive (CNSA, https://db.cngb.org/cnsa/) under CNP0001954. The processed data for human genetic variants and microbiome associations are uploaded in Figshare under 10.6084/m9.figshare.c.6719184.v1. The computer codes for the analyses in this study are uploaded in GitHub under https://github.com/VincentBioinfo/COPD_genetics/.
