# Supplementary material for: Human genetic associations of the airway microbiome in chronic obstructive pulmonary disease
Source: Respir Res. 2024 Apr 16;25:165. doi: 10.1186/s12931-024-02805-2 (PMC11367891; doi:10.1186/s12931-024-02805-2)
Supplement: Supplementary file 1 — Supplementary Material 1. [file 12931_2024_2805_MOESM1_ESM.docx]

**Human genetic associations of the airway microbiome in chronic obstructive pulmonary disease**

Supplementary document


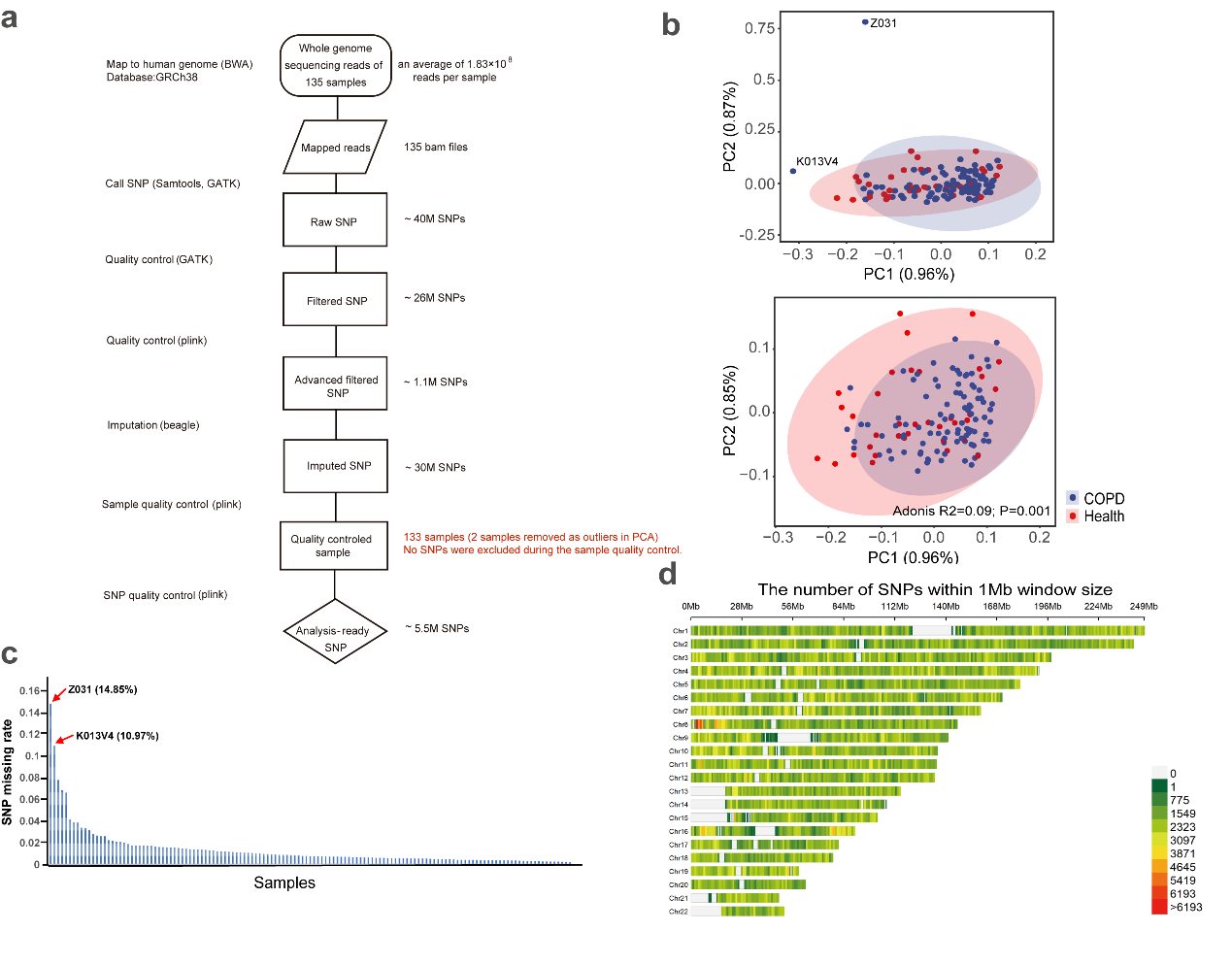


**Figure S1. Overview of the SNP calling process and the genetic variation of 135 individuals. a)** Flow diagram showing the SNP calling, sample quality control, and SNP filtering steps. **b)** Principal component analysis (PCA) plots of the 135 samples based on genetic variation. Two clear outliers were marked by their sample IDs and excluded in downstream analysis. PCA plots were also showed when removing the two outliers. **c)** SNP missing rate of all 135 samples, with the two outlier samples being the highest. **d)** SNP density plot across the 22 human chromosomes showing the number of SNPs within 1 Mb window size. The horizontal axis represents the chromosome length in Mb. The colors correspond to SNP density.


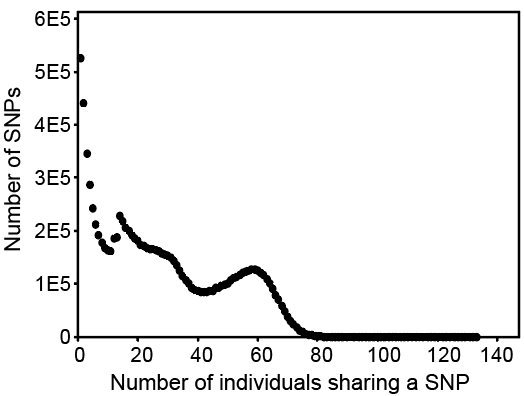


**Figure S2.** SNP sharing across individuals. Shown are the number of SNPs as a function of the number of individuals that share them.


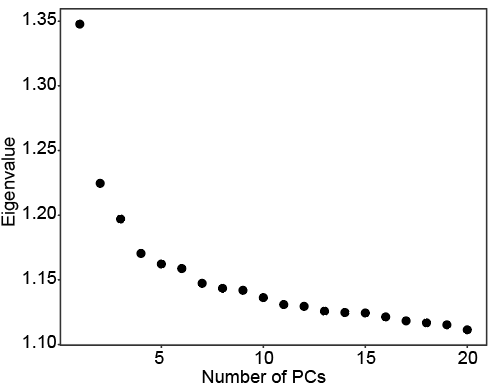


**Figure S3.** The distribution of the first 20 PCs from the PCA of the host genetic data. Eigenvalue of each of the top 20 PCs was plotted.


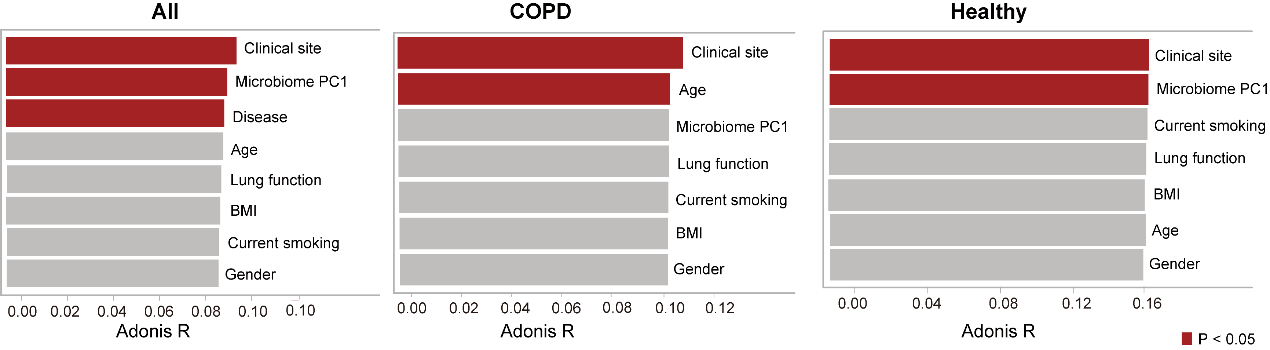


**Figure S4.** The results for a ‘reverse’ PERMANOVA associating microbiome PC1 and other demographic and clinical features with the host genetic profiles.


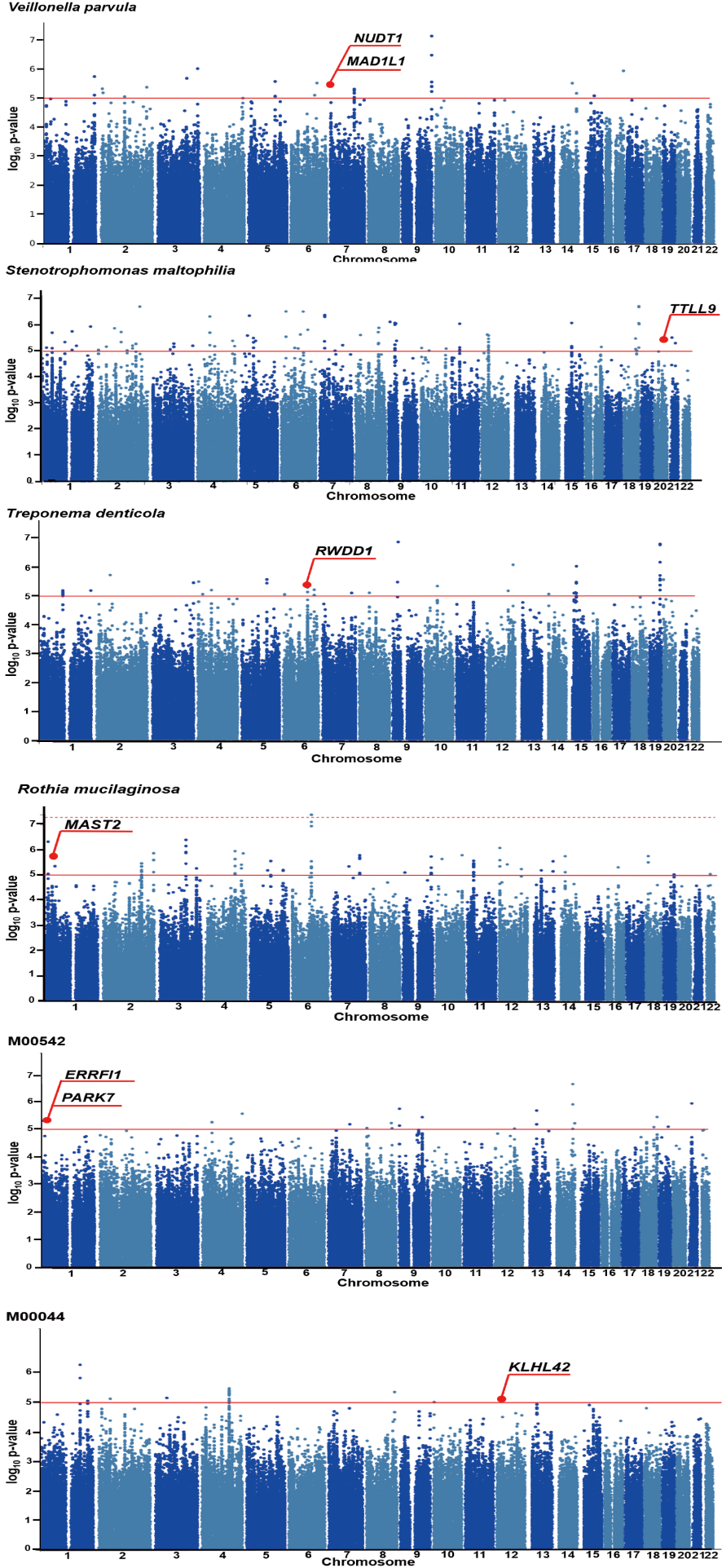


**Figure S5.** Manhattan plots showing the genetic associations of *Veillonella parvula*, *Stenotrophomonas maltophilia*, *Treopnema denticola*, *Rothia mucilaginosa*, functional module M00542 and M00044 and their corresponding host genes and SNPs.

**
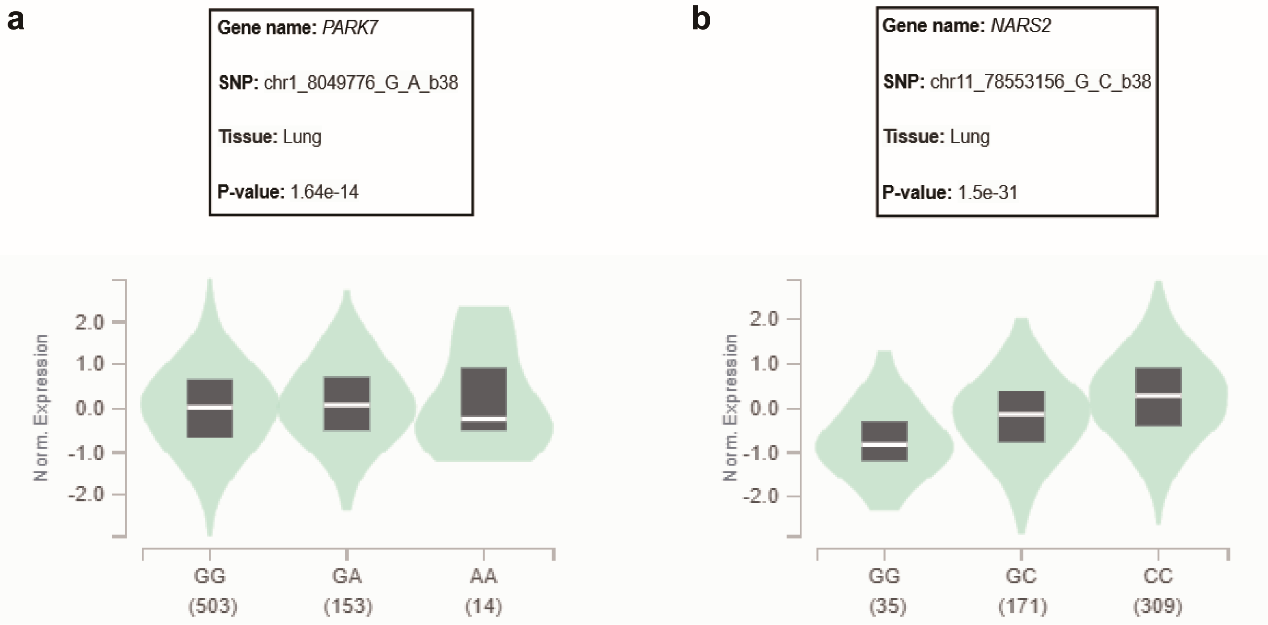
**

**Figure S6.** The eQTL plots showing relationships between gene expression levels in the lung tissue and the genetic variation of their corresponding SNPs in GTEx database for *PARK7* and *NARS2*.


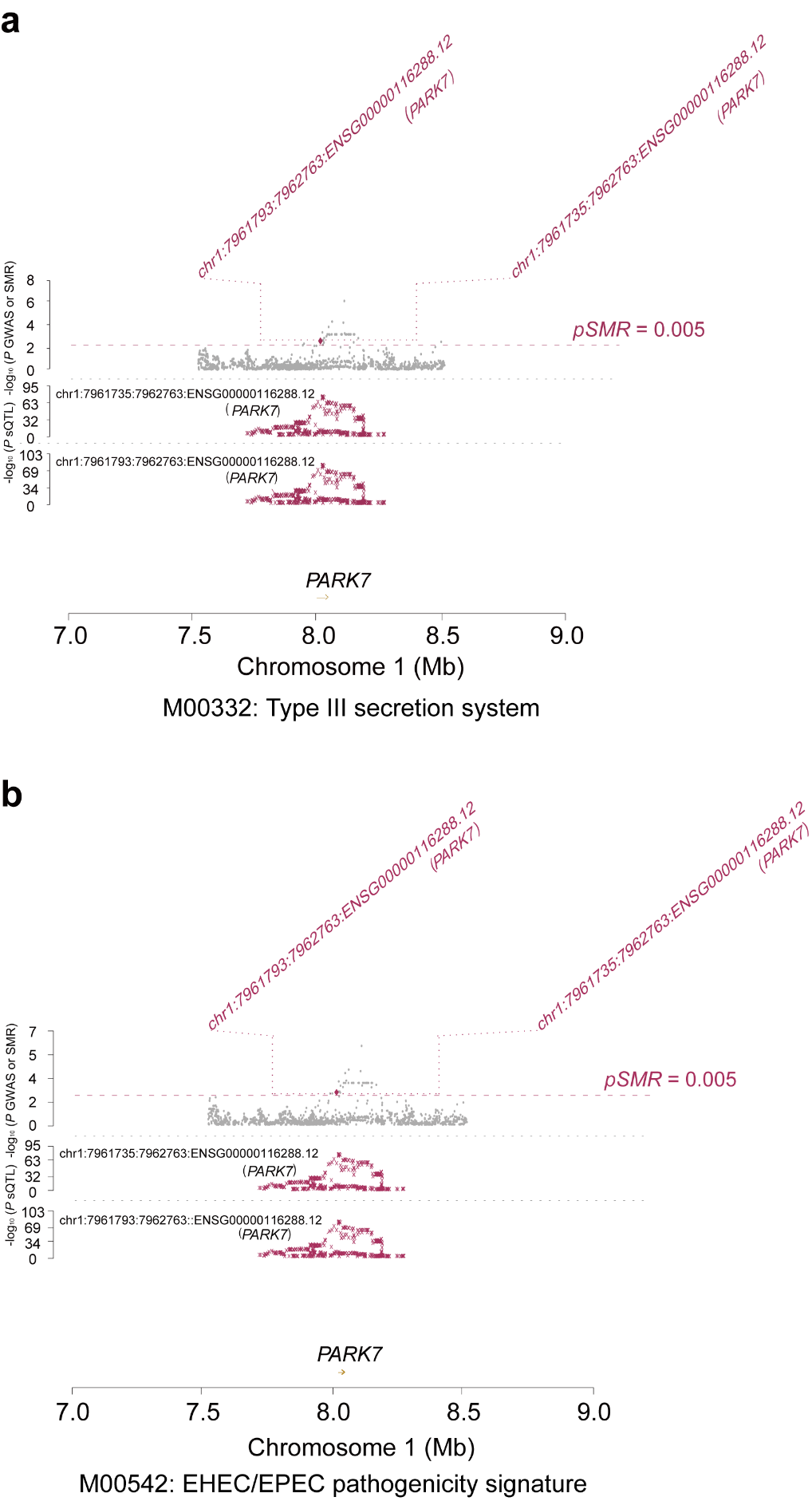


**Figure S7. SMR-based associations between sQTL and microbiome functional modules. a-b)** The manhattan plots show *P*-values of SNPs in association with two KEGG modules (M00332 and M00542). The bottom plots represent *P*-values of SNPs in association with gene splicing level probes in SMR test.


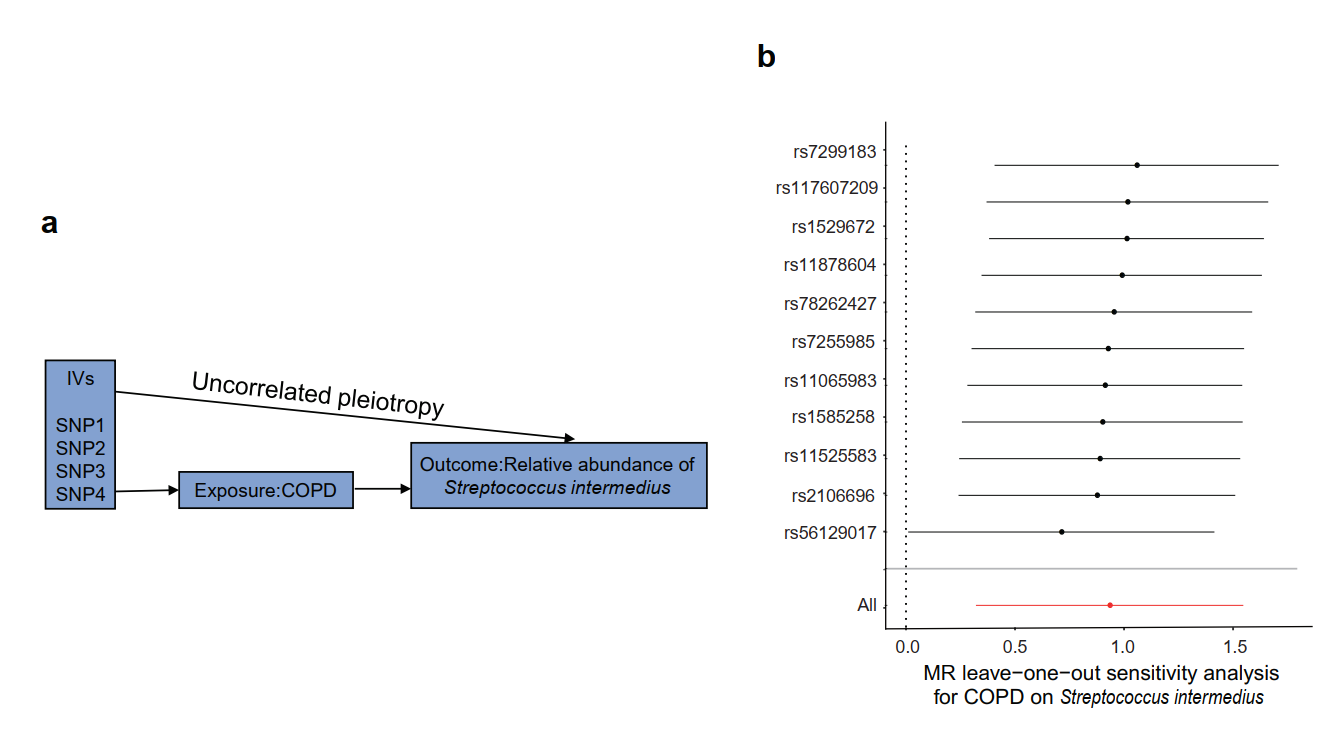


**Figure S8. Diagram of genetic causality inference and the results of leave-one-out sensitivity analysis. a)** MR analysis using SNPs as instrumental variables for estimating the influence of COPD as exposure on the microbiome as outcome (i.e. *S. intermedius*). **b)** MR leave-one-out sensitivity analysis for the effect of COPD on *S. intermedius.*

**Supplementary Tables**

**Table S1. Demographic features for COPD patients and healthy controls in this study.** Shown are demographic factors (age, gender, smoking), clinical features (GOLD, exacerbation frequency, long-term antibiotics, ICS, spirometry, CAT score, mMRC score), sputum and blood cell counts.

**Table S2.** **Detailed information on the list of significant genes associated with lung function (FEV1/FVC, FEV1%), sputum neutrophil percentage (NEU), sputum eosinophil percentage (EOS), GOLD status, ICS usage, and smoking status.** The table shows the rsIDs, positions, associated genes and GWAS *P*-values of their independent significant SNPs. Positional mapping is performed in FUMAGWAS, based on ANNOVAR annotations by specifying the maximum distance between SNPs and genes or based on functional consequences of SNPs on genes. The mapped genes highlighted in red with a function descriptor in brackets are those genes that were reported as genetically associated with the corresponding phenotype in existing databases (Open Targets Genetics, GWAS Catalog, and GWASATLAS), suggesting independent functional validation. The associations reaching genome-wide significance (P<5×10^-8^) are highlighted in bold and yellow background. chr: chromosome, pos: position.

**Table S3.** **Detailed information on host genetic associations of the selected 52 species and 106 functional modules.** The table shows the phenotypes, the number of candidate SNPs, the number of mapped genes, the number of COPD-associated genes and their gene symbols. Also shown are the SNPs reaching genome-wide significance (P<5×10^-8^), their P-values, and the mapped genes. The genes that were transcriptionally linked to the microbiome features (as shown in Table 1) are highlighted in red.

**Table S4.** **Detailed information on the 113 COPD-related genes and their genetic associations with microbiome features.** The table shows the rsIDs, positions, associated genes and *P*-values of their independent significant SNPs. The details of the previously reported COPD-associated genes, including the study PMID, lead variant, and association P-value, are also shown. Also shown are the Spearman correlations (significant associations highlighted in red) of the microbiome features and host genes and key COPD clinical traits (neutrophil percentage, eosinophil percentage, smoking, ICS usage, GOLD status). The genes transcriptionally linked to the microbiome features (as shown in Table 1) are highlighted in yellow background.

**Table S5. Detailed information for host genetic associations of all 517 microbiome species and 461 functional modules.** The table shows the phenotypes, the number of candidate SNPs, the number of mapped genes, the number of COPD-associated genes and their gene symbols.

**Table S6.** **Detailed information on the microbiome-host genetic associations that reached genome-wide significance for the expanded set of 517 species and 416 functional modules (*P*<5.0x10^-8^).** The table shows the rsIDs, positions, associated genes and association *P*-values of their independent significant SNPs. The COPD-associated genes are highlighted in red.

**Table S7. Detailed information on microbiome association with 1,427 SNPs with genome-wide significant association with COPD/lung function from 19 datasets in public domain.** The table shows detailed information for 19 GWAS catalog datasets, the detailed publication records (including the first author, journal, publication date, study population, PMID, title), the rsIDs, position, associated genes and *P*-values of their independent significant SNPs in association with microbiome taxa and functional modules. The associations of these SNPs with the selected microbiome features (52 taxa and 106 functional modules), as mentioned in the main text, are highlighted in yellow background.

**Table S8. Detailed information for host genetic associations of two *Lactobacillus* species (*Lactobacillus salivarius* and *Lactobacillus oris*).** The table shows the rsIDs, position, associated genes and *P*-values of their independent significant SNPs in association with the two species. The most significant associations, as mentioned in the main text, are highlighted in yellow background.

**Table S9.** **Detailed information on SMR analysis.** The table shows 7 SMR associations including 3 eQTL SMR and 4 sQTL SMR with *P*-value passing the threshold (*P*<0.0033 for taxa and *P*<0.0042 for module). Shown are the rsIDs, positions, se, beta, P-values in GWAS, eQTL/sQTL and SMR analyses, and P-values and the number of SNPs implemented in HEIDI test. A1: minor allele, A2: major allele, freq: genotype frequency, se: standard error, GWAS: genome-wide association study, eQTL: expression quantitative trait loci, SMR: summary data-based Mendelian randomization, HEIDI: heterogeneity in dependent instruments.

**Table S10. Detailed information on the instrumental variable used in bidirectional MR analysis.** A total of 25 instrumental variables (11 forward and 14 reverse) were implemented in MR analysis. Shown are the rsIDs, positions, allele information, F-statistics, eaf, P-values, se, and beta in exposure and outcome GWAS summary. chr: chromosome, pos: position, eaf: effect allele frequency, beta: beta coefficient, se: standard error.

**Table S11.** **Detailed information on the associations between *Streptococcus intermedius* and COPD in MR heterogeneity and pleiotropy tests.** Shown are Q, degree of freedom, and P-value in heterogeneity test, and egger intercept, se, and P-value in pleiotropy test. MR: Mendelian randomization, IVW: inverse variance weighted, Q: Cochran's Q statistic, Q df: degree of freedom of Cochran's Q statistic, se: standard error, Q pval: P-value in Cochran's Q statistic, se: standard error, pval: P-value of the pleiotropy test.

**Table S12. List of bacterial species flagged as potential contaminants by being present in at least two of four reagent controls (relative abundance>0.001).** Also shown are their average abundances in the sputum samples in COPD and controls in both sites.

**Table S13. STROBE-MR checklist of recommended items of the two-sample Mendelian randomization studies.**
